# Single-Cell Transcriptomics of Regulatory T Cells Reveals Trajectories of Tissue Adaptation

**DOI:** 10.1016/j.immuni.2019.01.001

**Published:** 2019-02-19

**Authors:** Ricardo J. Miragaia, Tomás Gomes, Agnieszka Chomka, Laura Jardine, Angela Riedel, Ahmed N. Hegazy, Natasha Whibley, Andrea Tucci, Xi Chen, Ida Lindeman, Guy Emerton, Thomas Krausgruber, Jacqueline Shields, Muzlifah Haniffa, Fiona Powrie, Sarah A. Teichmann

**Affiliations:** 1 Wellcome Sanger Institute, Wellcome Genome Campus, Hinxton, UK; 2 Centre of Biological Engineering, University of Minho, Braga, Portugal; 3 Kennedy Institute of Rheumatology, Nuffield Department of Orthopaedics, Rheumatology and Musculoskeletal Sciences, University of Oxford, Oxford, UK; 4 Translational Gastroenterology Unit, Experimental Medicine Division Nuffield Department of Clinical Medicine, University of Oxford, John Radcliffe Hospital, Oxford, UK; 5 Institute of Cellular Medicine, Newcastle University, Newcastle-Upon-Tyne, UK; 6 MRC Cancer Unit, University of Cambridge, Cambridge, UK; 7 Centre for Immune Regulation and Department of Immunology, University of Oslo and Oslo University Hospital, 0372 Oslo, Norway; 8 Theory of Condensed Matter, Cavendish Laboratory, Department of Physics, University of Cambridge, Cambridge, UK; 9 European Molecular Biology Laboratory, European Bioinformatics Institute (EMBL-EBI), Wellcome Genome Campus, Hinxton, UK

## Abstract

Non-lymphoid tissues (NLTs) harbor a pool of adaptive immune cells with largely unexplored phenotype and development. We used single-cell RNA-seq to characterize 35,000 CD4^+^ regulatory (Treg) and memory (Tmem) T cells in mouse skin and colon, their respective draining lymph nodes (LNs) and spleen. In these tissues, we identified Treg cell subpopulations with distinct degrees of NLT phenotype. Subpopulation pseudotime ordering and gene kinetics were consistent in recruitment to skin and colon, yet the initial NLT-priming in LNs and the final stages of NLT functional adaptation reflected tissue-specific differences. Predicted kinetics were recapitulated using an *in vivo* melanoma-induction model, validating key regulators and receptors. Finally, we profiled human blood and NLT Treg and Tmem cells, and identified cross-mammalian conserved tissue signatures. In summary, we describe the relationship between Treg cell heterogeneity and recruitment to NLTs through the combined use of computational prediction and *in vivo* validation.

## Highlights

• scRNA-seq reveals Treg cell tissue-specific signatures
• The phenotype of Treg cells shows progressive adaptation to barrier tissues
• Treg cells share transcriptional dynamics of adaptation to colon and skin
• Modules of peripheral Treg cell identity are conserved between mouse and human

## Graphical Abstract

**Figure:**
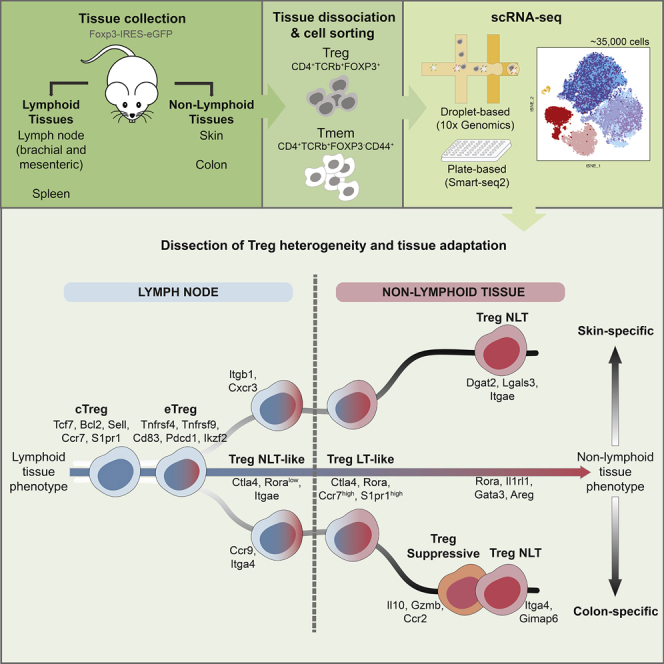

## Teaser

Treg cells are known to display tissue-specific heterogeneity. Miragaia and colleagues probe Treg cells in lymphoid and barrier tissues by single-cell transcriptomics. They show that Treg cells segregate into subpopulations along a continuum of tissue adaptation and present conserved expression programs between homeostasis and disease and mouse and human.

## Introduction

Regulatory T (Treg) cells are a specialized CD4^+^ T cell subset that controls immune responses and play a central role in homeostasis (Sakaguchi, 2004, Izcue et al., 2009). Recent studies have described unique tissue-specific adaptations of non-lymphoid tissue (NLTs) Treg cells distinct from their lymphoid tissue (LT) counterparts. This includes acquisition of an effector phenotype with expression of transcripts encoding effector molecules (*Ctla4*, *Gzmb*, *Klrg1*), chemokines and their receptors (*Ccr4*), and immunosuppressive cytokines (*Il10*) (Panduro et al., 2016, Bollrath and Powrie, 2013), in addition to tissue-specific signature genes associated with their role in each environment (Liston and Gray 2014). Nonetheless, their full transcriptional phenotype and its reflection on NLT population heterogeneity is yet to be uncovered.

Trafficking of T cells to NLTs occurs in steady-state conditions and development (Kimpton et al., 1995, Thome et al., 2016), as well as in response to harmless stimuli at barrier surfaces such as commensal bacteria and dietary antigens (Ivanov et al., 2008). Treg cell migration requires tissue-specific cues involving integrins, chemokine, and other G-protein coupled receptors (Cepek et al., 1994, Kim et al., 2013, Chow et al., 2015).

To provide a deeper insight into Treg cell populations in NLTs, we analyzed single-cell RNA-seq (scRNA-seq) data of Treg cells from mouse colon and skin and compared them to LT populations. We identified various transcriptionally distinct clusters of Treg cells in LTs and NLTs, namely a subpopulation in the LTs, which showed heavy priming to the NLT environment. Pseudotime ordering of these subpopulations further revealed the transcriptomic adaptations occurring in Treg cells during their transition from the lymph node to barrier tissues. Our results show that these steady-state adaptations share a core signature between bLN-to-skin and mLN-to-colon trajectories, indicative of a general NLT residency program in barrier tissues. These findings were recapitulated during *de novo* Treg cell recruitment to melanoma in a murine model system. Lastly, we examined the evolutionarily conservation of NLT Treg cells’ identity between mouse and human.

## Results

### Treg and Tmem Cell Identity in NLTs Is Driven by a Common Expression Module

**Figure 1:**
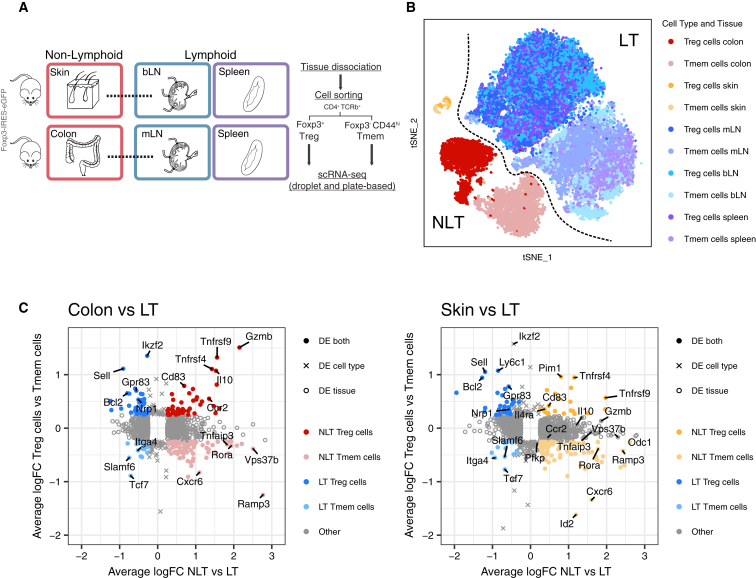
Steady-State scRNA-Seq Datasets of CD4^+^ T Cells from LT and NLT (A) Experimental design for scRNA-seq data collection. (B) t-SNE representing all Treg and Tmem cells that passed quality control. (C) Genes defining the identity of Treg and Tmem cells in lymphoid and non-lymphoid tissues. Colon and skin were individually compared with their corresponding draining lymph node and spleen cells. See also Figure S1.

We performed scRNA-seq on isolated CD4^+^Foxp3^+^ (Treg) and CD4^+^Foxp3^-^CD44^high^ memory (Tmem) T cells (Figure S1A) from two barrier NLT sites—the colonic lamina propria (hereinafter referred to as colon) and the skin—their lymphoid counterparts in the draining mesenteric and brachial lymph nodes (mLN and bLN), and the spleen from a Foxp3-GFP mouse reporter line (Bettelli et al., 2006) (Figure 1A). We will refer to Treg and Tmem cells together as CD4^+^ T cells. For each sorted population, single-cells were captured using the droplet-based microfluidic system Chromium (10× Genomics), hereinafter referred to as 10×. We obtained 30,396 good quality cells (see Experimental Procedures, Figure S1C, Table S1). Using the same gating strategy, two Smart-seq2 (Picelli et al., 2014) plate-based datasets were produced independently. These confirmed findings drawn from the 10× and complemented them with higher gene coverage and full T cell receptor (TCR) sequences.

A tSNE projection (Figure 1B) after filtering (Figure S1B; Table S2) showed a division between LT and NLT, with cells from LTs divided into two clusters, according to cell-type. NLT cells formed one single skin cluster and two clusters separating Treg and Tmem cells from colon (Figure 1B). We defined gene-expression signatures for Treg and Tmem cells in peripheral tissues by examining differentially expressed (DE) genes between all NLT and LT cells and, in parallel, between Treg and Tmem cells (Figure 1C). NLT T cell populations are characterized by the expression of several elements of the TNFRSF-NF-κB pathway, including transducers (*Traf1*, *Traf4*, *Traf2b*), effectors (*Nfkb1*, *Nfkb2*, *Rel*, *Rela*, *Relb*), and inhibitors (*Nfkbib*, *Nfkbid*, *Nfkbie*). In Tmem cells, these were accompanied by cytokines (*Tnfsf8*, *Tnfsf11*) and various pathway inhibitors, such as *Tnfaip8*. In contrast, NLT Treg cells expressed TNF receptors (*Tnfrsf4*, *Tnfrsf9*, *Tnfrsf18*) and transducers (*Pim1*), underscoring the importance of signaling via the TNFRSF-NF-κB axis in controlling Treg cells in the peripheral tissues. Several chemokine receptors appeared DE across tissues and cell types. *Ccr4*, *Ccr8*, and *Cxcr4* were upregulated in both colon and skin T cells, while *Ccr1* and *Ccr5* were specific to colon and *Ccr6* to skin. *Cxcr6* was more highly expressed in NLT Tmem cells. We also detected other genes involved in NLT identity (*Crem*, *Rgs2*, *Il1r2*, *Icos*, *Hif1a*, *Kdm6b*, *Gata3*), including some specific to Tmem (*Vps37b*, *Id2*, *Ramp3*, *Tnfsf8*) and Treg cells (*Il10*, *Gzmb*, *Ctla4*, *Cd83*, *Socs2*).

Together, the scRNA-seq datasets collected provide a comprehensive overview of Treg and Tmem cells in multiple lymphoid and non-lymphoid tissues and identify the TNFRSF-NF-κB pathway as key to their barrier tissue identity.

### Heterogeneity within LT and NLT Treg Cell Populations Reflects Distinct Degrees of Commitment to the Peripheral Phenotype

**Figure 2:**
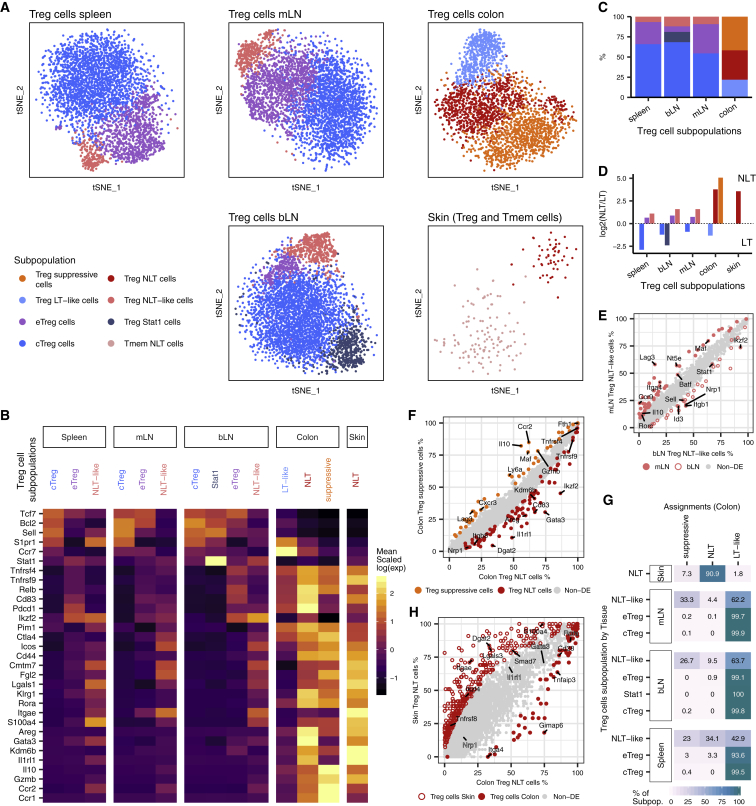
Heterogeneity within LT and NLT Treg Populations (A) t-SNE projections of Treg cells per tissue, colored by subpopulation. cTreg, central Treg; eTreg, effector Treg. (B) Subpopulation marker gene mean expression (*Z* score). Values greater than 2.5 or lower than −1.5 are colored equally. (C) Relative proportions of Treg cell subpopulations within each tissue that revealed heterogeneity. (D) NLT/LT signature score in each Treg cell subpopulation, measured as the ratio between the number of NLT and LT genes that have been identified as significantly upregulated in each cluster. (E) Percentage of cells expressing each gene in Treg NLT-like cells from mLN and bLN. Genes that are upregulated in the bLN subpopulation are represented by an open circle, and genes upregulated in mLN are represented by a filled circle. (F) Percentage of cells expressing each gene in colon Treg suppressive and Treg NLT subpopulations. (G) Matching of non-colonic Treg cells to colonic Treg cell subpopulations using a logistic regression model (90% accuracy, see Experimental Procedures). Table shows the percentage of each identified subpopulation (y axis) that were labeled by the model as each Treg cell cluster (x axis). (H) Percentage of cells expressing each gene in skin Treg NLT and colon Treg NLT cell subpopulations. See also Figure S2.

Treg cell phenotypical and functional heterogeneity has been extensively discussed in recent years (Josefowicz et al., 2012, Campbell and Koch, 2011). Clustering our data within each tissue grouped Treg cells into distinct subpopulations (Figure 2A) with clearly defined marker genes (Figure 2B; Table S3). Across lymphoid organs, we identified central and effector Treg (cTreg and eTreg) cell subsets (Cretney et al., 2011, Vasanthakumar et al., 2015). cTreg cells express typical LT-associated markers, such as *Tcf7*, *Bcl2*, *Sell*, *S1pr1*, while eTreg cells expressed a subset of NLT-associated genes, like *Tnfrsf9*, *Relb*, *Ikzf2*, and *Pdcd1*. We also detected a subpopulation of Treg cells with high expression of *Stat1* and interferon-stimulated genes exclusively in the bLN. A fourth, less frequent population in lymphoid tissues (∼5%–10%; Figure 2C), which we named Treg NLT-like cells, expresses eTreg cell markers, as well as genes characteristic of NLT T cells, such as *Itgae*, *Rora*, *Fgl2*, and *Klrg1* (Figure 2B). We hypothesize that this population is primed to migrate and adapt to NLTs. Indeed, DE genes between NLT-like Treg cells from mLN and bLN revealed that the colon-homing molecules *Ccr9* and *Itga4*, as well as their regulator *Batf* were upregulated specifically in the mLN, while *Cxcr3* and *Itgb1* were present in the bLN (Figure 2E). These differences were not observed between other LN subpopulations (data not shown).

To quantify the bias toward LT or NLT phenotypes, we calculated an NLT-LT marker gene signature for each cluster (Figure 2D; see Experimental Procedures). Consistently across all LTs, cTreg cells exhibited a clear LT signature, while eTregs and NLT-like Tregs leaned toward an NLT profile, which was more pronounced in the latter.

In the colon, we found three subpopulations of Treg cells that we labeled as NLT, suppressive and LT-like. Treg NLT and suppressive cells were present in equal proportions, both exhibiting NLT traits (Figures 2C and 2D). Treg NLT cells in colon express higher amounts of *Gata3*, *Nrp1*, *Areg*, *Il1rl1*, and *Ikzf2*, matching the known thymic-derived GATA3^+^-subpopulation (Schiering et al., 2014)(Hu and Zhao 2015) while suppressive colonic Treg cells expressed more *Il10*, *Gzmb*, *Lag3*, and *Cxcr3*, resembling the peripherally-derived RORγt^+^-subpopulation (Ohnmacht et al., 2015, Schiering et al., 2014, Sefik et al., 2015). *Rorc* itself, while not present as a marker, appears in a higher percentage of Treg suppressive cells (6.16% versus 2.85% in colonic Treg NLT cells). Technical limitations for detection of lowly expressed genes by scRNA-seq might account for the difficulty in capturing *Rorc* transcripts. Lastly, LT-like Treg cells differed from other colonic populations by expressing LT-associated genes including *Sell*, *Ccr7*, *Tcf7*, and *Bcl2*, and lower amounts of NLT-associated genes such as *Klrg1*, *Cd44*, *Icos*, *Rora*, *Tnfrsf9*, and *Itgae* (Figure 2B).

In contrast to the colon, and likely as a consequence of fewer cells captured, skin Treg cells did not show evident heterogeneity (Figure 2A). They expressed an unequivocal NLT signature (Figure 2D), but it was not clear to which colonic Treg cell populations they were most similar (Figure 2B). We addressed this by using a logistic regression model to calculate the probability of each skin Treg cell identifying as one of the colonic subpopulations (Figure 2G, see Experimental Procedures). This revealed that most skin Treg cells were more similar to colonic Treg NLT than to Treg suppressive cells. Accordingly, colon Treg NLT cell marker genes *Gata3*, *Il1rl1*, *Tnfrsf4*, and *Rora* were not differentially expressed between skin and colon Treg NLT cells (Figure 2H, Figure S2A). Despite their resemblance, differences in function and/or state between skin and colon Treg NLT might reside in a few genes. Among these are *Dgat2*, an enzyme involved in lipid synthesis in skin (Fagerberg et al., 2014), and *Ikzf4*, a transcription factor relevant for Treg stability (Sharma et al., 2013).

The same approach classified most central and effector Treg cells from spleen, mLN, and bLN (Figure 2G) as colonic Treg LT-like cells. Treg NLT-like cells from these lymphoid tissues, on the other hand, were more similar to Treg NLT and Treg-suppressive cell populations in the colon. Both the mLN and the bLN had a higher proportion of Treg cells assigned as suppressive than spleen, which contained the highest fraction of Treg NLT cells. We confirmed the presence and proportions of Treg cell subpopulations in the Smart-seq2 datasets by matching these cells to the subpopulations found across LTs and NLTs in the 10× dataset (Figure S2B).

Clustering of Tmem cells revealed multiple subpopulations (T helper-1 [Th1 cell], Th2 cells, Th17 cells, T follicular helper [Tfh] cells, lymphoid) (Figures S2C and S2D; Table S3) distributed differently across the tissues analyzed (Figure S2D). Th1, Th2, and Th17 cells in lymphoid tissues exhibited a stronger NLT phenotype than Tmem lymphoid cells and Tfh cells (Figure S2E), which is likely an indication of their ability to adapt to and function in the NLTs.

In summary, scRNA-seq allowed us to dissect the heterogeneity of Treg cells from LTs and NLTs. We identified NLT- and LT-like Treg cell subpopulations that suggest progressive cross-tissue adaptation to the NLT environment. We found a close correspondence between skin and colonic Treg NLT cells, while revealing differences in gene expression that might explain their adaptation to the two environments.

### Adaptation of Treg Cells to Skin and Colon Relies on a Shared Transcriptional Trajectory

The mechanisms underlying Treg cell recruitment and adaptation from LT to NLT are far from understood. Having identified multiple subpopulations at different stages of NLT adaptation (Figure 2D), we further dissected the dynamics of this transition.

We obtained evidence of CD4^+^ T cell recruitment from LT to NLT by reconstructing TCR clonotypes using TraCeR (Stubbington et al., 2016) from the Smart-seq2 datasets. This showed Tmem and Treg cell clones present in LNs and respective NLTs (Figures S3A and S3B), suggesting cell migration between them.

**Figure 3:**
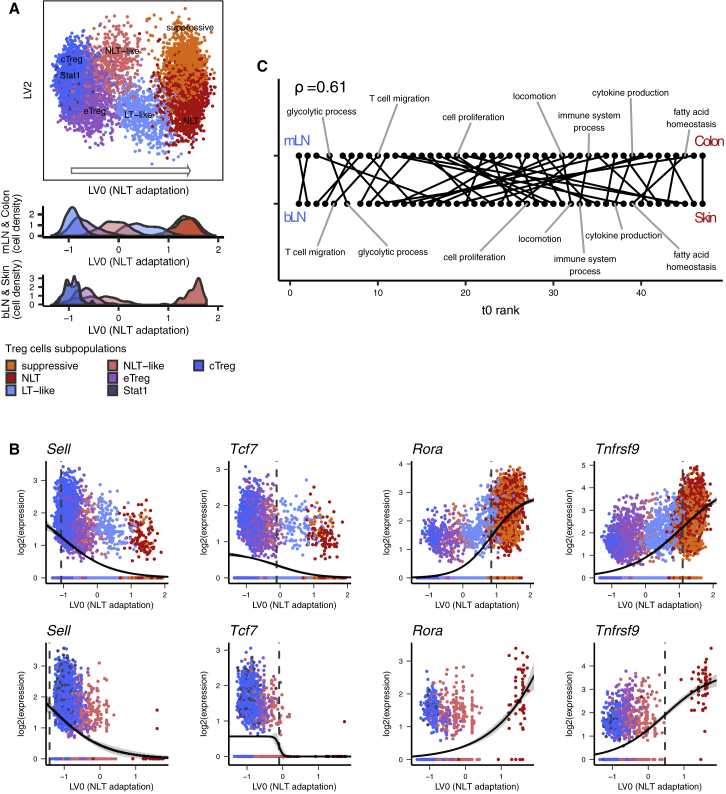
Reconstruction of Treg Cell Recruitment from Lymphoid to Non-Lymphoid Tissues in Steady-State (A) Top two latent variables (LV) found with BGPLVM for mLN and colonic Treg cells, with bLN and skin Treg cells mapped over the same coordinates. LV in the x axis is the most relevant one, and mapping of colon and skin subpopulations over it reveals a transition of Treg cell identity across tissues. (B) Gene expression in mLN and colon (top) or bLN and skin (bottom) over LV0 modeled into a sigmoidal curve. Dashed vertical line marks the activation point of each gene. (C) Sequence of activation of GO biological processes across the transition to colon (top) or skin (bottom), evidencing a conservation between both trajectories (Spearman’s rho - 0.61). See also Figure S3.

To identify Treg cell LN-to-NLT adaptation trends in the data, we reconstructed a pseudospace relationship between cells by obtaining latent variables (LV) from Bayesian Gaussian Process Latent Variable Modeling (BGPLVM, see Experimental Procedures) (Michalis et al., 2010). Along the mLN to colon trajectory laid out by LV0, Treg cells are ordered from cTreg to eTreg cells, followed by NLT-like and LT-like Treg cells, and ending with the overlapping Treg suppressive and Treg NLT cell subpopulations (Figure 3A, “Colon” density plot, Figure S3C). This order matches the increasing expression of NLT marker genes and decrease of LT ones across mLN subpopulations (Figures 2B and 2D). Importantly, Treg NLT-like cells from the mLN partially mixed with Treg LT-like cells from the colon, supporting the notion that NLT adaptation is a continuous process spanning LT and NLT. Overall, LV0 accurately represented the progressive migration and adaptation of Treg cells to the NLT environment, providing a reference to study the gene expression dynamics along this process. Skin and bLN Treg cells were projected onto the latent space defined for colon and mLN, resulting in a similar subpopulation distribution (Figure 3A, “Skin” density plot; see Experimental Procedures). Nevertheless, a similar projection was observed when using just those cells (Figures S3C and S3D). Applying the same approach to the Smart-seq2 datasets yielded similar distributions of the inferred cell subpopulations (Figure S2B) along the LT-to-NLT adaptation trajectory, as well as considerable overlaps between LV correlated genes (Figures S3E–S3H).

The use of velocyto (La Manno et al., 2018) to infer the directionality of adaptation suggests that most Treg cells found in the NLTs, as well as some of the NLT-like Treg and eTreg cells, are adapting toward a more pronounced NLT phenotype (Figure S3I).

We then used the inferred LN-NLT trajectory to identify the cascade of transcriptional changes driving adaptation to NLTs by modeling genes with a sigmoid curve and find their activation or deactivation “times” (Figure 3B; Table S4; see Experimental Procedures). We found 812 and 1209 genes with a switch in expression (either up or down) along the bLN-to-skin and mLN-to-colon trajectories, respectively, with 511 of those being shared. LT-related genes (*Lef1*, *Tcf7*, *Sell*) were downregulated, while NLT associated genes like *Nfil3*, *Ccr8*, *Cxcr6*, *Gzmb* were upregulated. TNFRSF-NF-κB-related genes (*Tnfrsf1b*, *Tnfrsf4*, *Tnfrsf18*) and the *Batf* transcription factor were upregulated still in the LN, reflecting the relevance of this pathway for eTreg cell development and the NLT phenotype (Vasanthakumar et al., 2017, Vasanthakumar et al., 2015). Toward the NLT side of the trajectory there is evidence of further Treg cell differentiation, with upregulation of additional genes involved in this pathway (*Nfkb2, Tnfrsf9*), as well as other effector molecules (*Il10*, *Cd44*). Important regulators for the final tissue adaptation include *Rora*, recently described in skin Treg cells (Malhotra et al., 2018). We searched for enriched Biological Processes GO Terms, and calculated the mean time of activation or deactivation (t0) of the genes within each term. We found the gene expression kinetics along the adaptation trajectories to skin and to colon to be consistent (Spearman’s rho = 0.61, Figure 3C): T cell migration and glycolytic process are among the earlier events in both colon and skin, followed by cell proliferation; cytokine production and fatty acid homeostasis emerge toward the end of the adaptation trajectory.

In summary, we determined a continuous trajectory aligning Treg cell subpopulations from bLN, mLN, skin, and colon according to the stage of recruitment and adaptation to the NLT environments. Furthermore, the consistent ordering of gene expression programs shows that gene kinetics leading to NLT adaptation follows a similar regulatory sequence in both bLN-to-skin and mLN-to-colon trajectories.

### Treg Cell Recruitment into Steady-State Skin and Melanoma Tumors Uses Common Mechanisms

**Figure 4:**
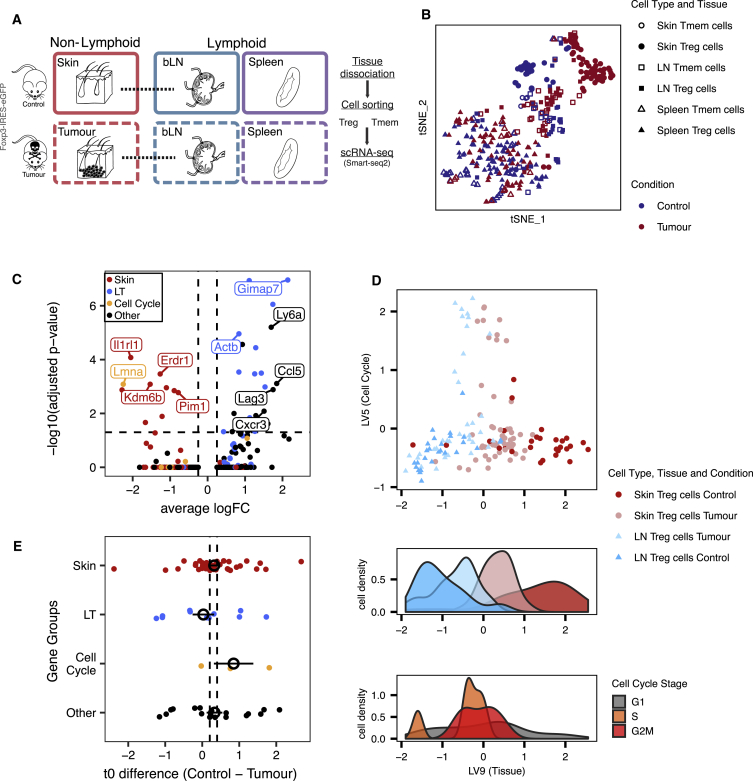
Recruitment and Adaptation of Treg Cells to the Tumor Environment Recapitulates Steady-State Migration (A) Melanoma induction strategy and sampled tissues. (B) t-SNE depicting Treg and Tmem cells from tumor and steady-state skin, draining brachial lymph nodes, and spleen. (C) Differential expression between skin and tumor Treg cells. Treg cells classified as cycling were excluded. (D) (top) Latent variables found with MRD-BGPLVM representing cell cycle (LV5) and non-lymphoid tissue recruitment/adaptation of Treg cells (LV9). (bottom) Distribution of cells based on Tissue and Condition and Cell-Cycle phase along the recruitment trajectory. (E) Difference in activation time (t0) of genes in control and tumor. Genes are classified as being markers of skin, lymph node, cell cycle, or other. Colored points show mean ± mean SE for each group. Vertical dashed lines represent the mean ± SE for all t0 values. t test between control and melanoma t0 indicates no change (p value = 0.2631), with t0 values having a Spearman correlation coefficient of 0.65 between both conditions. See also Figure S4.

To validate our findings in steady-state cells, we used a mouse melanoma model to investigate whether Treg cell migration and adaptation trajectory to peripheral tissues could be recapitulated. Previous studies analyzing human TCR repertoires (Sherwood et al., 2013, Plitas et al., 2016) have shown that tumor-Treg cells are likely to be recruited *de novo* from LTs and not from the adjacent NLT, despite exhibiting a phenotype similar to that of NLT Treg cells (Plitas et al., 2016, De Simone et al., 2016). We therefore purified Treg and Tmem cells from B16.F10 melanomas or PBS controls 11 days after subcutaneous implantation into Foxp3-IRES-eGFP reporter mice (Haribhai et al., 2007) to produce a plate-based scRNA-seq dataset (Figure 4A; see Experimental Procedures).

Skin and tumor Treg cells clustered separately (Figure 4B). As with steady-state skin, we observed shared clonotypes between tumor and bLN Treg cells (Figure S4B). In the tumor-bearing mice, we detected an additional cluster of cycling cells in both the LN and tumor (Figure S4A). These observations suggest *de novo* recruitment from LN and simultaneous expansion in both tumor and draining-LN. DE between non-cycling tumor Treg and control skin Treg cells revealed a relatively small number of genes significantly different between the two Treg cell populations (28 upregulated in tumor and 10 in steady-state skin; Figure 4C), in line with recently published human data (Plitas et al., 2016). Tumor Treg cells upregulate the exhaustion marker *Lag3* (Malik et al., 2017), as well as *Cxcr3* and *Ccl5*, while control skin Treg cells upregulate skin Treg cell markers such as *Il1rl1*, *Pim1*, *Sdc4*, *Kdm6b*, and *Erdr1*. However, skin Treg cell signature genes such as *Batf*, *Tnfrsf4*, *Tnfrsf9*, *Samsn1*, *Tigit*, *Tchp*, *Ccr8*, *Ccr2*, and *Itgav* are similarly expressed in both populations.

Next, we sought to obtain a shared migration trajectory of steady-state versus perturbed system (tumor model) Treg NLT cells recruitment. To this end, we used the MRD-BGPLVM algorithm (Damianou et al., 2012) (see Experimental Procedures) to explore gene expression trends across Treg cells from the control skin, tumor, and respective draining-LNs together. Two main latent variables were identified, one explained almost entirely by cell-cycle-associated variability (LV5), and one mainly associated with the LT-NLT signature (LV9) (Figure 4D, Figure S4C). Notably, NLT adaptation trajectory (LV9) was strongly related to the trajectories found in control and melanoma conditions when MRD-BGPLVM is applied to each one individually (respectively, 86% and 61% of genes correlated with LV9 are also correlated with control LV1 and tumor LV1; Figures S4E–S4H, see Experimental Procedures).

Gene kinetics along NLT adaptation (LV9) for each condition show 158 shared genes, with 71% of which also present in the steady-state skin trajectory determined previously. Values of t0 remain largely unchanged between control and melanoma (Figure 4E), suggesting that NLT recruitment and adaptation follow the same program in homeostatic and perturbed conditions. The tissue adaptation genes shared between control and melanoma include many of the players in the TNFRSF-NF-κB pathway we previously described in the steady-state (*Tnfrsf9*, *Tnfrsf18*). These were accompanied by genes associated with cell migration and adhesion (*Ccr2*, *Gpr55*, *Plxna2*), transcription factors (*Rora*, *Ikzf3*, *Id2*, *Batf*, *Hif1a*, *Prdm1*), secreted factors (*Lgasl1*), and others related to immune activation and effector states (*Klrg1*, *Icos*, *Tigit*, *Gzmb*).

Despite the similarities between melanoma and control trajectories, cells from both conditions do not completely overlap, and Treg cells could be ordered by NLT adaptation between populations (from least to most adapted cells: control LN, melanoma LN, tumor, and control skin) (Figure 4D). This implies that in response to an immune challenge in a barrier tissue, a higher fraction of Treg cells in the LNs acquires NLT adaptations. In fact, for several NLT markers we observed more cells expressing them in the tumor-draining LN compared to the control, e.g., *Id2* (59% versus 26%), *Batf* (57% versus 26%), and *Lgals1* (89% versus 67%), further supporting our hypothesis that there is priming of Treg cells to NLTs while still in the LN. Overall, Treg cells from challenged mice recapitulate the steady-state NLT adaptation.

### The Core Identity of NLT Treg Cells Is Conserved between Mouse and Human

**Figure 5:**
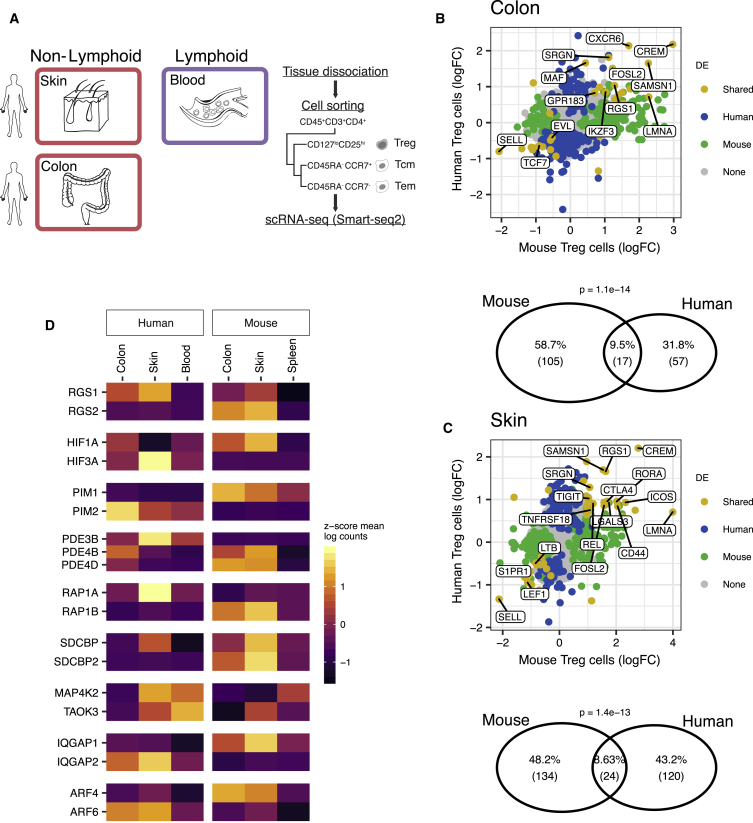
Human-Mouse Comparison of NLT Treg Cell Marker Genes (A) Tissues and cell types sampled from human. (B and C) Top shows overlap between NLT Treg cell markers detected in human and mouse, in either (B) colon or (D) skin datasets. Bottom shows fold-change between gene expression in non-lymphoid and lymphoid tissues in mouse and human. Blood and spleen were used as lymphoid tissues in human and mouse, respectively. (D) NLT paralogs exhibiting opposing expression patterns between human and mouse. See also Figure S5.

We complemented our characterization of murine NLT Treg and Tmem cells by collecting human Treg cells, as well as Tmem (sorted into central and effector memory) cells from blood and skin, and from tumor-adjacent colon sections from patients undergoing colonic resection (Figure 5A, Figure S5). Similar to the mouse analysis, we identified gene markers for human CD4^+^ T cell populations (see Experimental Procedures).

Focusing on one-to-one orthologs, we found that 24 out of 144 human skin Treg cell markers and 17 out of 74 human colon Treg cell markers overlapped with the respective mouse signature. In colon, we observe the conservation of *Tnfrsf4*, *Lgals1*, *Srgn*, *Cxcr6*, *Maf*, or *Ikzf3* (Figure 5B), genes that we had previously identified as important in defining tissue identity and Treg cell subpopulations. The same applied to skin Treg cells, where we saw expression of *Batf*, *Rora*, *Rel*, *Srgn*, *Tnfrsf18*, and *Tigit* across species (Figure 5C). Overall, this indicates a conserved role of the core NLT signature, namely the TNFRSF-NF-κB-pathway.

In several instances, we observed the expression pattern of one gene being substituted by a paralog in the other organism (Figure 5D). For example, while the kinase *Pim1* is a marker of mouse NLT Treg cells and was not expressed in human, the inverse was true of *Pim2*. A similar situation was observed for *Rgs1-Rgs2*, *Hif1a*-*Hif3a*, and others. This suggests that some paralogous proteins have evolved to substitute each other during evolution of NLT Treg cells in mammals. The fact that several of the identified cases are receptors related to signal transduction leads us to believe that evolution of cell-cell communication pathways owes some plasticity to differential paralog usage.

Our cross-species comparison suggests that despite cross-species differences, the NLT Treg cell adaptation program defined in mouse is generally conserved in human.

## Discussion

Our work sheds light on the phenotype of skin and colon Treg cells. We profiled NLT Treg and Tmem cells to identify global relationships between cell populations, discriminating general CD4^+^ and specific Treg cell markers in NLT. We found that these Treg populations conserve fundamental traits shared across the skin and colon compartments, namely a substantial prevalence of genes part of the TNFRSF-NF-κB axis.

We leveraged the single-cell resolution of our data to explain Treg cell heterogeneity in the context of LT-to-NLT transition. Besides the eTreg cell state previously described in lymphoid organs (Cretney et al., 2011), we found two transitional subpopulations, Treg NLT-like cells in the lymphoid tissues and Treg LT-like cell in the non-lymphoid ones, which together explain the cross-tissue transition from central Treg to Treg NLT cell populations. NLT-like Treg cells in the mLN and bLN showed extensive NLT-priming, including the upregulation of tissue-specific homing-molecules to the drained NLT. Others have demonstrated that a subpopulation of spleen Treg cells can express a partial visceral adipose tissue (VAT) signature and later give rise to fully-mature VAT-Treg cells upon migration (Li et al., 2018), implying that this is valid for various tissues and should be considered in the design of future precision medicine strategies involving targeting of Treg cells to NLTs.

Our pseudotime results support migration and adaptation relationships between subpopulations and allowed us to explore the basic mechanisms for the establishment of peripheral Treg cell phenotypes. In this transition, metabolic and proliferation changes in Treg cells happen concurrently with priming for migration, followed by changes in cytokine production machinery upon establishment in the periphery. Despite the overall similarity of recruitment and adaptation to NLTs, and although all three subpopulations (skin NLT, colon NLT, colon suppressive) fell close along the NLT adaptation trajectory, colon but mainly skin Treg NLT cells exhibited greater adaptation to the NLT environment. We hypothesize that the upregulation of *Ikzf4*, *Dgat2*, and *Itgae* observed in skin might explain and contribute to the further stabilization, retention, and metabolic adaptation of Treg cells to the NLT compartment.

Treg cell priming in LNs is apparent from their increased NLT signature and expression of tissue-homing molecules, yet it is likely that Treg NLT-like cells are a heterogeneous subpopulation, with some cells egressing to the NLTs and others recently drained from the NLTs. This was confirmed using velocyto and agrees with the bidirectional migration between LNs and the NLTs described in skin using a photoconversion system (Matsushima and Takashima 2010). Studies coupling photoconversion and scRNA-seq can further our understanding of Treg cell migration patterns, as previously shown with single-cell qPCR (Ikebuchi et al., 2016).

A considerable proportion of the adaptation program between bLN-to-tumor was contained within the bLN-to-skin trajectories. Similar to steady-state, cues derived from NLTs are likely to prime Treg cells located in the draining LNs, as indicated by a higher percentage of cells expressing *Batf*, *Lgals1*, *Id2*, and other NLT markers in melanoma. In sum, tumor Treg cells resemble less mature versions of their homeostatic skin counterparts that, nevertheless, follow the same NLT adaptation trajectory.

Despite the conserved tissue-specific signatures, the differential paralog usage we identified between species suggests a pivotal role for expanded gene families in rewiring signaling pathways throughout evolution. Studies focusing not only on tissue-resident cells, but also on the surrounding environment and organs can help dissect the relevance of these pathways in T cell biology and how this evolutionary rewiring might affect immune response and homeostasis.

Overall, we reveal a dynamic adaptation of T cells as they traffic across tissues and provide an open resource (http://www.teichlab.org/data/) for investigating *in vivo* CD4^+^ T cell phenotypes in mouse and human, to ultimately harness NLT CD4^+^ T cells as future therapeutic targets.

## STAR★Methods

### Key Resources Table

**Table:** 

REAGENT or RESOURCE	SOURCE	IDENTIFIER
**Antibodies**		

anti-CD8a	BioLegend	Cat# 100714; RRID: AB_312753
anti-CD19	BioLegend	Cat# 115530; RRID: AB_830707
anti-CD11b	BioLegend	Cat# 101226; RRID: AB_830642
anti-TCRb	BioLegend	Cat# 109218; RRID: AB_493346
anti-CD4	BioLegend	Cat# 100553; RRID: AB_2561388
anti-CD44	BD Biosciences	Cat# 563970; RRID: AB_2738517
anti-CD62L	BioLegend	Cat# 104426; RRID: AB_493719
anti-ST2	MD Biosciences	Cat# 101001B; RRID: AB_947551
anti-CD45	BD Biosciences	Cat# 557833; RRID: AB_396891
anti-CD3	BD Biosciences	Cat# 557694; RRID: AB_396803
anti-CD4	BD Biosciences	Cat# 557922; RRID: AB_396943
anti-CD8	BioLegend	Cat# 300924; RRID: AB_1575074
anti-CD25	BioLegend	Cat# 302606; RRID: AB_314276
anti-CD127	BioLegend	Cat# 351309; RRID: AB_10898326
anti-CCR7	BioLegend	Cat# 353226; RRID: AB_11126145
anti-CD45RA	BD Biosciences	Cat# 563031; RRID: AB_2722499

**Biological Samples**		

Human blood and normal breast tissue	Breast reduction plastic surgeries (REC approval number: 08/H0906/95+5)	N/A
Human colonic normal tissue adjacent to tumor	Colon resection surgeries (NHS Research Ethic System reference number: 11/YH/0020)	N/A

**Chemicals, Peptides, and Recombinant Proteins**		

RPMI 1640 Medium	GIBCO	Cat#21870076
Bovine serum albumin (BSA)	Sigma-Aldrich	Cat#A9418
HEPES	GIBCO	Cat#15630080
Collagenase D	Roche	Cat#11088866001
Liberase TL	Roche	Cat#5401020001
Percoll	GE Healthcare	Cat#17-0891-01
EDTA	Invitrogen	Cat#AM9260G
Collagenase VIII	Sigma-Aldrich	Cat#C2139
Fetal calf serum (FCS)	Sigma-Aldrich	Cat#F4135
Collagenase A	Roche	Cat#10103583001
DNase I	Roche	Cat#11284932001
Collagenase IV	Worthington-Biochemical	Cat#LS004188
DTT (for digestion)	Sigma-Aldrich	Cat#D0632
DMSO	Sigma-Aldrich	Cat#D2650
Penicillin-Streptomycin-Glutamine	GIBCO	Cat#10378-016
Lymphoprep	STEMCELL Technologies	Cat#07801
DAPI	Sigma-Aldrich	Cat#D9542
96-well plates	4titude	Cat#F4135
RNase Inhibitor	Clontech	Cat#2313A
Triton X-100	Sigma-Aldrich	Cat#T9284
dNTPs	ThermoFisher	Cat#10319879
ERCC RNA Spike-in mix	Ambion	Cat#4456740
SMARTScribe™ Reverse Transcriptase	Clontech	Cat#639538
AMPure XP beads	Beckman Coulter	Cat#A63881

**Critical Commercial Assays**		

Chromium single cell Chip kit V2	10x Genomics	Cat#120236
Chromium single cell 3′ Library and Gel Bead kit V2	10x Genomics	Cat#120237
Agilent High Sensitivity DNA kit	Agilent	Cat#5067-4626
Nextera XT DNA Sample Preparation kit	Illumina	Cat#FC-131-1096

**Deposited Data**		

Smart-seq2 raw data	This paper	ArrayExpress: E-MTAB-6072
10x Chromium raw data	This paper	ArrayExpress: E-MTAB-7311
Processed count matrices and metadata	This paper	https://figshare.com/projects/Treg_scRNA-seq/38864
Interactive data browser	This paper	www.teichlab.org/data

**Experimental Models: Cell Lines**		

Melanoma: B16.F10	ATCC	CRL-6475

**Experimental Models: Organisms/Strains**		

Mouse: Foxp3-IRES-GFP	The Jackson Laboratory	JAX: 006772

**Oligonucleotides**		

Oligo-dT	(Picelli et al., 2014); IDT	N/A
Template Switch Oligo (TSO)	(Picelli et al., 2014); Exiqon	N/A
ISPCR	(Picelli et al., 2014); IDT	N/A

**Software and Algorithms**		

R	https://www.r-project.org/	Version: 3.5.1; RRID: SCR_001905
RStudio	https://www.rstudio.com/	Version: 1.2.720
CellRanger	10x Genomics	Version: 1.2.0

**Other**		

Illumina Nextera XT protocol sample preparation protocol	Fluidigm	PN 100-5950 B1
10x library preparation protocol	10x Genomics	https://support.10xgenomics.com/single-cell-gene-expression/library-prep/doc/user-guide-chromium-single-cell-3-reagent-kits-user-guide-v2-chemistry
Plate-based scRNA-seq (Smart-seq2)	(Picelli et al., 2014)	N/A

### Contact for reagents and resource sharing

Further information and requests for resources and reagents should be directed to the Lead Contact, Sarah A. Teichmann (st9@sanger.ac.uk).

### Mice

All mice were maintained under specific pathogen-free conditions at the Wellcome Genome Campus Research Support Facility (Cambridge, UK) and at the Kennedy Institute for Rheumatology (Oxford, UK). All procedures were in accordance with the Animals Scientific Procedures Act 1986. For steady-state experiments, the Foxp3-GFP-KI mouse reporter line (Bettelli et al., 2006) was used. The melanoma challenge was performed in Foxp3-IRES-GFP genetically targeted reporter mice (Haribhai et al., 2007) purchased from The Jackson Laboratory (stock no. 006772). In both cases, 6-14 week-old mice were used.

### Human samples

Human skin and blood samples were obtained from patients undergoing breast reduction plastic surgeries (REC approval number: 08/H0906/95+5). Surgical-resection specimens were obtained from patients attending the John Radcliffe Hospital Gastroenterology Unit (Oxford, UK). These specimens were obtained from normal regions of bowel adjacent to resected colorectal tumors from patients undergoing surgery. Informed, written consent was obtained from all donors. Human experimental protocols were approved by the NHS Research Ethics System (Reference number:11/YH/0020). Further details concerning patients and tumors can be found in Table S5.

### Isolation of murine leukocytes for steady-state skin dataset

To isolate leukocytes from ear tissue, ears were removed at the base, split into halves and cut into very small pieces. Tissue was digested in 3.5ml RPMI medium (GIBCO) with 0.1% BSA, 15mM HEPES, 1mg/mL collagenase D (Roche) and 450μg/mL Liberase TL (Roche) for 60 min at 37°C in a shaking incubator at 200rpm. Digested tissue was passed through an 18G needle to further disrupt the tissue and release cells. Cells were filtered through a 70μm cell strainer, and the digestion was terminated by addition of ice-cold RPMI containing 0.1% BSA (Sigma-Aldrich) and 5mM EDTA (Invitrogen). A three-layer (30, 40, 70%) Percoll (GE Healthcare) density-gradient was used to enrich for the lymphocytes. Cells obtained from the digestion were layered in the 30% layer on top of the 40% and 70% layers, and centrifuged for 20 min at 1800rpm without brake. Cells at the 40/70% interface were collected for the subsequent analysis. Cell suspensions from spleen and bLN were prepared as described previously (Uhlig et al., 2006).

### Isolation of murine leukocytes for steady-state colon dataset

Colons were washed twice in RPMI medium (GIBCO) with 0.1% BSA (Sigma-Aldrich) and 5mM EDTA (Invitrogen) in a shaking incubator at 200rpm at 37°C to remove epithelial cells. The tissue was then digested for an hour in RPMI with 10% FCS, 15mM HEPES (GIBCO) and 100U/mL collagenase VIII (Sigma-Aldrich). Digestion was terminated by addition of ice-cold RPMI with 10% FCS (Sigma-Aldrich) and 5mM EDTA (Invitrogen). Leukocyte enrichment and suspension was obtained as described in the previous paragraph.

### Melanoma induction and cell isolation

The melanoma induction experiments were performed in accordance with UK Home Office regulations under Project License PPL 80/2574. The protocol used was adapted from a previous publication (Riedel et al., 2016). For syngeneic tumors, 2.5 × 10^5^ B16.F10 melanoma cells (ATCC) were inoculated subcutaneously into the shoulder region of 6- to 14-week-old female Foxp3-IRES-GFP mice (Haribhai et al., 2007). Animals were excluded if tumors failed to form or if health concerns were reported. Control Foxp3-IRES-GFP mice were injected with 50 μl PBS. Animals were culled after 11 days. Tumors, tumor-draining (brachial) lymph nodes and spleen were isolated for subsequent analysis. PBS-injected and steady-state skin, draining lymph nodes (bLN) and spleen were collected from control mice. Tumor and PBS-injected skin were mechanically disrupted and digested in a 1ml mixture of 1 mg/mL collagenase A (Roche) and 0.4 mg/mL DNase I (Roche) in PBS (solution A) at 37°C for 1h with 600rpm rotation. 1ml of PBS containing 1mg/mL Collagenase D (Roche) and 0.4 mg/mL DNase I (Roche) (solution B) was then added to each sample, which returned to 37°C for 1h with 600 rpm rotation. Lymph nodes were digested for 30min in 500μl of solution A, and for further 30min after the addition of 500μl of solution B. EDTA (Invitrogen) at the final concentration of 10mM was added to all samples. Spleens were processed as described previously (Uhlig et al., 2006). Suspensions were passed through a 70 μm cell strainer before immunostaining. Samples from different animals were kept separated throughout processing and sorting.

### Isolation of human CD4^+^ T cells

#### Isolation of leukocytes from human skin

Plastic surgery skin included reticular dermis to the depth of the fat layer. The upper 200 microns of skin were harvested using a split skin graft knife. Whole skin was digested in RPMI 1640 with 100IU/mL penicillin, 100ug/mL streptomycin, 2mM L-glutamine (GIBCO), 10% FCS (Sigma-Aldrich) and 1.6mg/mL type IV collagenase (Worthington-Biochemical) for 12-16 hours at 37°C and 5% CO_2_. Digest was passed repeatedly through a 10ml pipette until no visible material remained. To yield a single-cell suspension, digest was passed through a 100-micron filter into a polypropylene sorting tube.

#### Isolation of leukocytes from human colon

Normal regions of bowel adjacent to resected colorectal tumors were prepared as previously described, with minor modifications (Bettelli et al., 2006, Geremia et al., 2011). In brief, mucosa was dissected and washed in 1 mM dithiothreitol (DTT) (Sigma-Aldrich) solution for 15 min at room temperature to remove mucus. Specimens were then washed three times in 0.75 mM EDTA (Invitrogen) to deplete epithelial crypts and were digested for 2h in 0.1 mg/mL collagenase A solution (Roche). For enrichment of mononuclear cells, digests were centrifuged for 30 min at 500 g in a four-layer Percoll (GE Healthcare) gradient and collected at the 40%/60% interface.

#### Peripheral blood mononuclear cell isolation

10mL blood from skin donors were collected into EDTA (Invitrogen). Density centrifugation with Lymphoprep (STEMCELL Technologies) was performed according to manufacturer’s instructions. Recovered cells were cryopreserved by pelleting and resuspending in 1ml heat-inactivated fetal calf serum containing 10% DMSO, and storing at −80°C.

Cryovials were later thawed in water bath, then rapidly being transferred to warmed medium (RPMI 1640 (GIBCO) with 100IU/mL penicillin, 100ug/mL streptomycin, 2mM L-glutamine (GIBCO), 10% FCS (Sigma-Aldrich)) and filtered through a 100-μm filter.

#### Flow cytometry and single-cell RNA sequencing

Mouse and human cell suspensions were stained with the antibodies in the Key Resource Table and DAPI (Sigma-Aldrich).

Droplet-based scRNA-seq datasets were produced using a Chromium system (10x Genomics), referred to as 10x. Cell populations of interest were sorted, manually counted, and their concentrations adjusted to enable the capture of ∼5000 cells (except for skin Treg and Tmem cells, for which we aimed to capture ∼300 each). The standard protocol for the 10x single cell 3′ kit (V2 chemistry) was followed and each cell population loaded onto a separate chip inlet. We ran each sample on one lane of Illumina HiSeq 4000, following manufacturer’s recommendations.

Two plate-based scRNA-seq datasets: the “colon dataset,” including Treg and Tmem cells from colon, mLN and spleen, and the “skin dataset” from skin, bLN and spleen. Single cells were sorted in 2μl of Lysis Buffer (1:20 solution of RNase Inhibitor (Clontech) in 0.2% v/v Triton X-100 (Sigma-Aldrich)) in 96 well plates, spun down and immediately frozen at −80°C. Smart-seq2 protocol (Picelli et al., 2014) was largely followed to obtain mRNA libraries from single cells. Oligo-dT primer, dNTPs (ThermoFisher) and ERCC RNA Spike-In Mix (1:50,000,000 final dilution, Ambion) were then added. Reverse Transcription and PCR were performed as previously published (Picelli et al., 2014), using 50U of SMARTScribe Reverse Transcriptase (Clontech). The cDNA libraries for sequencing were prepared using Nextera XT DNA Sample Preparation Kit (Illumina), according to the protocol supplied by Fluidigm. Libraries from single cells were pooled and purified using AMPure XP beads (Beckman Coulter). Pooled samples were sequenced on an Illumina HiSeq 2500 (paired-end 100-bp reads) or Illumina HiSeq 2000 v4 chemistry (paired-end 75-bp reads) aiming at an average depth of 1 million reads/cell.

#### RNA expression quantification

Sequencing data from 10x runs was aligned and quantified using the CellRanger software package with default parameters.

Gene expression from Smart-seq2 scRNA-seq data was quantified in counts using Salmon v0.6.0 (Patro et al., 2017), with the parameters–fldMax 150000000–fldMean 350–fldSD 250–numBootstraps 100–biasCorrect–allowOrphans–useVBOpt. For mouse, the cDNA sequences used contain genes from GRCm38 and sequences from RepBase, as well as ERCC sequences and an EGFP sequence. Since the EGFP RNA is transcribed together with Foxp3, counts from these two genes were added after quantification to represent Foxp3 expression. For human data quantification, cDNA sequences from GRCh38 and ERCC were used.

Standard scRNA-seq analysis (QC, differential expression and marker gene detection, and clustering) was performed using Seurat (Satija et al., 2015). All data was log-normalized using the NormalizeData function with a scale factor of 10000. Our expression data for different tissues is also available for user-friendly interactive browsing online at data.teichlab.org.

#### scRNA-seq quality control

Quality control of 10x-derived data was made taking into account number of UMIs - keeping cells with between 1000 and 15000 UMI - and number of genes - keeping cells with between 700 and 3500 genes with at least 1 UMI (Table S1). While cells were not filtered by their mitochondrial read content, cells with an elevated number of these reads are eventually removed via clustering (see “Subpopulation detection in 10x data”).

For Smart-seq2 data, count values for each cell were grouped in an expression matrix, and ERCC expression were separated from true gene expression. Cells were then filtered based on different quality parameters calculated for each dataset (Table S1). Additionally, the output of TraCeR (Stubbington et al., 2016) was used to remove cells without a detected TCR sequence, as well as invariant Natural Killer T (iNKT) cells and γ∂ T cells (defined as cells with at least one γ and one ∂ chain detected and no αβ pair). For the colon and skin datasets, 433 and 745 cells passed quality control, respectively.

Importantly, we note that TCR detection greatly improved our filtering by excluding cell types captured by FACS that did not fit the intended categories. This is the case for iNKT cells - captured mostly together with spleen T memory cells - and γδ-T cells - sorted together with skin Tmem cells in the melanoma experiment. Indeed, we also identified a NKT population in the 10x dataset, mostly within the cells sorted as spleen Tmem cells, as well as some LN Tmem cells (Figures S1B and S1C). We cannot, however, state that these are “invariant,” since we have no access to their complete TCR chains. TCR filtering also enables removal of cell doublets by identifying cells expressing an excessive diversity of recombined TCR chains. Even in cases of no allelic exclusion for TCR ɑ and β sequences, each cell would still only be able to produce two recombinants of each, allowing removal of cell doublets expressing more than two recombinants for a TCR locus. Lastly, we removed all cells not expressing any recombinant TCR in order to have a more stringent quality control. While in the human dataset the number of cells without a TCR was evenly distributed across tissues and cell types, there was a clear skew toward TCR absence in peripheral Treg cells (colon and skin) in the mouse datasets. These Treg cells did not appear to differ from the remaining population, having no differentially expressed genes or major differences in their overall number, presenting only a skew toward a higher number of reads (data not shown).

#### Dimensionality reduction methods

To obtain an overview of the datasets showing the relationships between cell population clusters, Principal Component Analysis (PCA) and tSNE were used. Before PCA, data was scaled using the ScaleData function (negative binomial model, normalizing by the number of UMI and centering the data). PCA and tSNE were calculated using the RunPCA and RunTSNE functions, respectively. For each dataset, a different number of Principal Components (PCs) and values for perplexity were used (Table S1), chosen by visual inspection of an elbow plot representing the relative importance of each PC. With exception of the PCA projection for the complete 10x dataset, only highly variable genes were used, calculated using the FindVariableGenes function from Seurat with the parameters num.bin = 100 and binning.method = ”equal_frequency.” Using all genes for dimensionality reduction of the whole 10x dataset resulted in more accurate clustering, allowing for the identification of most contaminant cells on this first step (Figure S1B). Plate-based datasets were treated separately as much as possible to avoid confounding batch effects from experiments performed separately.

#### Subpopulation detection in 10x data

To find clusters in the data, we used the FindClusters function from Seurat, with the same number of principal components used for tSNE. Cluster annotation was done by inspecting markers detected by the FindAllMarkers function.

Global clustering of the 10x dataset was done with the resolution parameter set to 0.2. After clustering the complete dataset, we excluded artifactual subpopulations (Figure S1, Table S2). A mixed Treg and Tmem cell population characterized by high expression of immediate-early response genes (e.g., *Jun*, *Junb*, *Fos*, *Fosb*), which has previously been reported in other cell types (Adam et al., 2017, van den Brink et al., 2017, Wu et al., 2017) was removed. An additional population of lymphoid tissue Tmem cells was also excluded because they presented expression profiles similar to NKT cells (*Nkg7*, *Ccl5*, *Cd160*, *Klrbc1, Cxcr6*).

Clustering on individual tissues used the following resolutions: for Treg cells, 0.3 on Spleen, 0.4 on bLN, 0.4 on mLN, 0.5 on Colon, 0.4 on all skin cells; for Tmem cells 0.4 on Spleen, 0.3 on bLN, 0.7 on mLN, and 0.6 on Colon. Annotation was performed and subpopulations characterized by immediate-early response genes were removed (Table S3).

#### Differential expression analysis

Differential expression (DE) and marker gene detection was performed using the FindMarkers and the FindaAllMarkers functions from the Seurat R package, using the default Wilcoxon test. Genes were considered differentially expressed if they had an average log fold-change of at least 0.25 and a Bonferroni-adjusted p value of 0.05 or lower.

For DE including all cells of the 10x dataset, a minimum of 5% of cells had to express the gene, otherwise a minimum of 1% was used. For comparisons between tests (for example Treg versus Tmem cells and LT versus NLT, see Figure 1C), the FindMarkers function was run twice - the first time to determine all genes considered expressed for each comparison, the second using the union of all those genes.

In the human and mouse comparison, human NLTs were compared to blood and mouse NLTs were compared to spleen only, and testing was restricted to genes with one-to-one orthologs.

#### Mapping cells to known populations using logistic regression classification

To make a correspondence of cells in the 10x dataset with the identified Treg cell subtypes in the colon (Figure 2G), or between Smart-seq2 data and the complete 10x dataset (Figure S2B), the counts and subpopulation labels of the 10x dataset Treg cell subpopulations and the complete 10x dataset were used to train a logistic regression classification model using scikit-learn with an L1 penalty and default parameters. The label with the highest probability predicted by the model was then attributed to each cell. The figures show the percentage of each tested population that was predicted as matching to each learned label.

#### Obtaining a migration latent variable for steady-state Treg cells

The large dimensionality of single-cell RNA-seq data has been used before to gain insights on time-dependent events (Trapnell et al., 2014, Lönnberg et al., 2017) by applying methods for pseudotime inference. Although it is impossible to follow one cell through the complete process, these methods can order single-cell data into a continuous dimension, using the discrete samples as snapshots containing a multitude of intermediate states.

Immune cells are expected to migrate between LTs and NLTs. We assumed that this effect would be reflected as a gradual single-cell expression phenotype, which could be captured as a latent variable of the data. To achieve this, we used Bayesian Gaussian Process Latent Variable Modeling (BGPLVM) (Michalis et al., 2010), implemented in the python package GPy (https://github.com/SheffieldML/GPy) as “GPy.models.BayesianGPLVM,” which was already used before for dimensionality reduction in scRNA-seq data to model Th1-Tfh cell differentiation (Lönnberg et al., 2017). BGPLVM was used on log-scaled counts and only considering highly variable genes. We run the method with six latent variables (LV) to be sure we capture the most relevant ones by Automatic Relevance Determination (ARD, Figure S3C), although this number does not alter significantly the performance of the algorithm. We then interpret the most important LV as the one ordering the cells between tissues along a migration and adaptation transition. In agreement, we observe gene expression changes associated with losing the lymphoid tissue identity and acquiring a peripheral tissue transcriptional profile (Figure 3B).

For 10x data, the method was used on mLN and colon Treg cells. We then mapped bLN and skin Treg cells onto the same LV using the predict function from the BGPLVM module, in order to have a similar coordinate system for both trajectories. Running BGPLVM with all data together would achieve a similar result (not shown). A BGPLVM projection of bLN and skin Treg cells (Figure S3D) shows an identical projection but with a wider gap between bLN and skin cells due to the large differences in cell numbers. We excluded spleen cells from this analysis to focus specifically on LN to NLT adaptation.

Similar effects are also observed in the corresponding Smart-seq2 cells (Figures S3F and S3G). We then show that all the LVs chosen as a “pseudospace variable” (LV0) have a similar effect between datasets by comparing the shared proportions of genes correlated with each of them (Figure S3H).

#### Identifying a common tissue migration trajectory in control and melanoma

Similarly to the steady-state, migration from the LN to the skin with a melanoma challenge is also expected. A common between-tissue Treg cell migration trajectory in control and melanoma conditions was obtained using Manifold Relevance Determination (Damianou et al., 2012) (MRD). MRD works by having an underlying BGPLVM model whose dimensions can be shared or private between sections of the data. Having the prior knowledge that a cell-cycle effect is present in the data (Figure S4A) and with the goal of obtaining a LV explaining tissue recruitment in both conditions, the melanoma dataset was divided into three sections for input: one with the expression in all cell-cycle associated genes, one with marker genes for any tissue, and one with the remaining genes. The importance of each section in each latent variable is shown in the ARD plot (Figure S4C). The model was run allowing for 12 LVs as output, and the one highly influenced by tissue-specific genes but not cell-cycle or other genes was used as a migration trajectory for both conditions (Figure 4D). The effects captured by these LVs can be observed in BGPLVM projections for the individual conditions (Figures S4E–S4G).

#### Switch-like genes in the migration latent variable

Gene expression changes in a continuous trajectory can be interpreted as a series of switch-like events. These can be modeled using a sigmoid curve, described by the following equation:

$$S=\frac{2\times \mu_{0}}{1+e^{-k\left(t-t_{0}\right)}}$$

where $\mu_{0}$is the mean expression between the sigmoid “on” and “off” states, $t_{0}$ is the point in which the switch in expression happens, and kdefines the sigmoid inclination and can be interpreted as the activation strength. Parameter kwill additionally inform on the direction of the switch (activation or inhibition) from its signal.

The R package switchde (Campbell and Yau, 2017) was used to model gene expression as a sigmoid in the inferred migration trajectories, using the appropriate latent variable as pseudotime.

In the steady-state 10x dataset partitions (mLN+colon Treg cells and bLN+skin Treg cells), switchde was applied for non-Tmem cell specific genes expressed in at least 30 cells, as well as genes with an absolute correlation greater than 0.25 with the LV chosen for both partitions. Due to the large differences in the number of cells in the skin partition, we ran switchde 100 times on different subsamples of each Treg cell subpopulation matching the smallest subpopulation size (405 for the colon partition, 55 for the skin partition), and used the median values of the parameters for further analysis. For the melanoma dataset, genes expressed in at least 5 cells in both conditions were tested. Only genes with a q-value ≤ 0.05 and that had a $t_{0}$within the LV range were kept for further interpretation.

#### RNA velocity estimation

RNA velocity is a measure that leverages detection of spliced and unspliced transcripts to predict single-cell development directionality (La Manno et al., 2018). We used velocyto to determine in which direction cells were changing in the cross-tissue adaptation trajectories. We have followed the python implementation of velocyto, and the code can be found in https://github.com/tomasgomes/Treg_analysis/blob/master/Velocyto.ipynb, where each of the runs is present.

#### Detection of expanded clonotypes

T cell receptor (TCR) sequences were reconstructed from single-cell RNA-seq data and used to infer clonality using TraCeR (Stubbington et al., 2016). We used TraCeR with the parameters–loci A B D G,–max_junc_len 120 to allow reconstruction of TCRα, TCRβ, TCR∂ and TCRγ chains in each cell and to permit TCRγ chains with long CDR3 regions.

#### GO Term enrichment

To test for enriched GO Biological Processes or KEGG Pathways in gene sets, the gprofiler R package (Reimand et al., 2016) was used, with the option of moderate hierarchical filtering enabled.

To determine the succession of Biological Processes GO Terms (Figure 3C, Table S4), we used the approach above on all genes called DE by switchde, and plotted only the terms with at least two genes.

#### Cell-cycle analysis

To assess potential effects of cell-cycle in the interpretation of the scRNA-seq datasets, Cyclone (Scialdone et al., 2015) (implemented in the scran R package) was used on all datasets. Results for the mouse melanoma dataset (where a relevant cycling population exists) were projected on the tSNE (Figure S4A). As the vast majority of cells was assigned to the default cell-cycle stage (G0/G1 in mouse, S in human), no cell-cycle correction was performed.

### Data Accessibility

scRNA-seq data for this project has been deposited in ArrayExpress under the accession numbers E-MTAB-6072 and E-MTAB-7311. Processed data can be found in https://figshare.com/projects/Treg_scRNA-seq/38864, and analysis notebooks can be found in https://github.com/tomasgomes/Treg_analysis.

## Supplemental Information

Document S1. Figures S1–S5

Table S1. Quality Control Criteria for Filtering Single Cell Transcriptomes in Each Dataset, Parameters for Dimensionality Reduction, and QC Rejection Fractions, Related to Figure 1 Cells were kept if they passed all these filters (see Experimental Procedures).

Table S2. Marker Genes for Low Resolution Clustering of All Droplet-Based scRNA-Seq Data, Related to Figure 1

Table S3. Marker Genes for Treg and Tmem Subpopulations found Per Organ, Related to Figure 2

Table S4. Genes Exhibiting Switch-like Behavior along NLT Adaptation Trajectories, Related to Figure 3

Table S5. Information on Human Donors with Biological Material Included in This Study, Related to Figure 5

Document S2. Article plus Supplemental Information
