# Supplementary material for: Single-Cell Transcriptomics of Regulatory T Cells Reveals Trajectories of Tissue Adaptation
Source: Immunity. 2019 Feb 19;50(2):493–504.e7. doi: 10.1016/j.immuni.2019.01.001 (PMC6382439; doi:10.1016/j.immuni.2019.01.001)
Supplement: Document S1. Figures S1–S5 [file mmc1.pdf]

Immunity, Volume *50*

Supplemental Information

Single-Cell Transcriptomics of Regulatory T Cells Reveals Trajectories of Tissue Adaptation

Ricardo J. Miragaia, Tomás Gomes, Agnieszka Chomka, Laura Jardine, Angela Riedel, Ahmed N. Hegazy, Natasha Whibley, Andrea Tucci, Xi Chen, Ida Lindeman, Guy Emerton, Thomas Krausgruber, Jacqueline Shields, Muzlifah Haniffa, Fiona Powrie, and Sarah A. Teichmann

Supplementary Table Legends

Table S1 - Quality control criteria for filtering single cell transcriptomes in each dataset, parameters for dimensionality reduction and QC rejection fractions. Cells were kept if they passed all these filters (see Methods). Related to Figure 1

Table S2 - Marker genes for low resolution clustering of all droplet-based scRNA-seq data. Related to Figure 1

Table S3 - Marker genes for Treg and Tmem subpopulations found per organ. Related to Figure 2

Table S4 - Genes exhibiting switch-like behaviour along NLT adaptation trajectories. Related to Figure 3

Table S5 - Information on human donors with biological material included in this study. Related to Figure 5

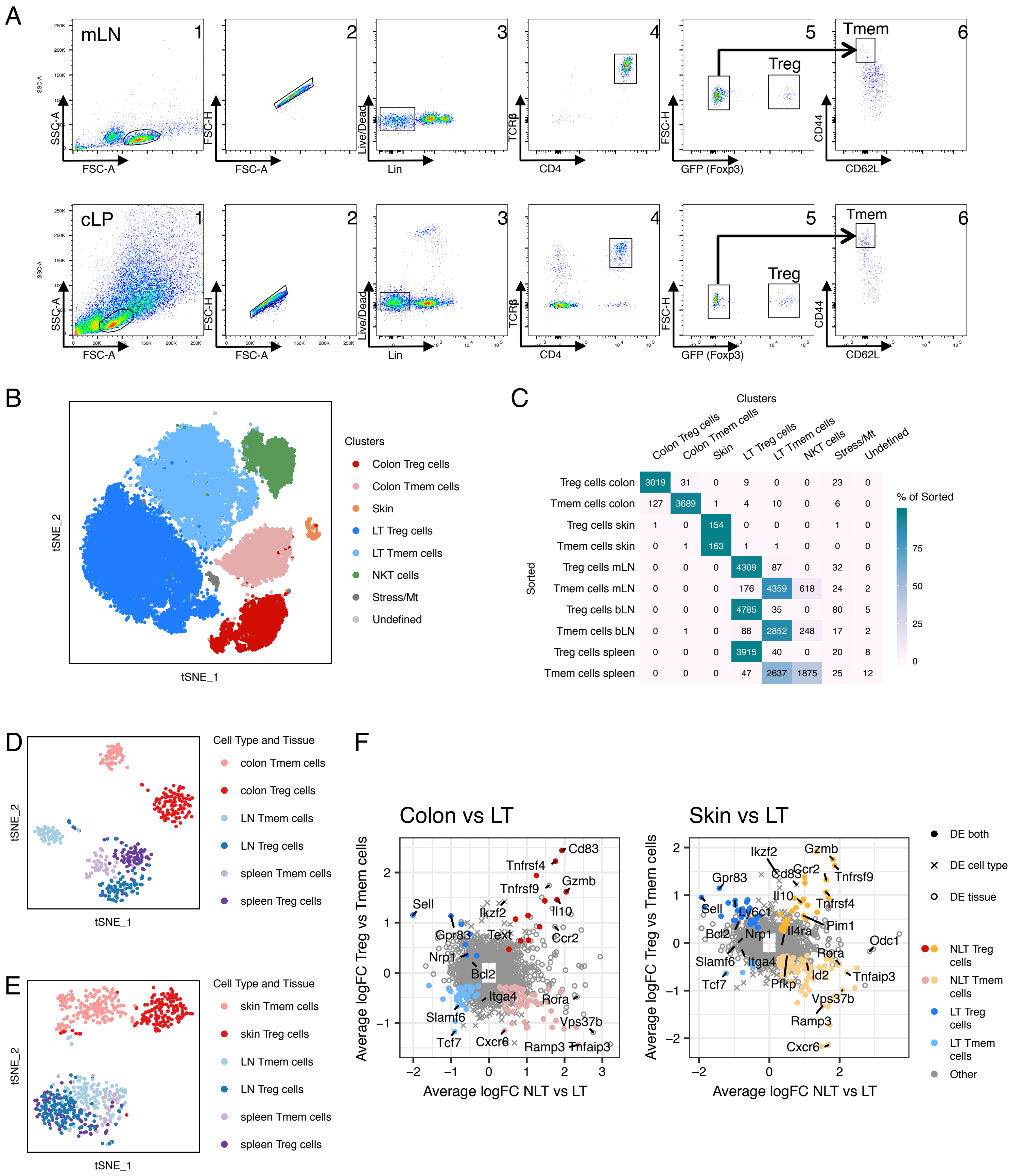

Sorted \ Clusters	Colon Treg cells	Colon Tmem cells	Skin	LT Treg cells	LT Tmem cells	NKT cells	Stress/Mt	Undefined
Treg cells colon	3019	31	0	9	0	0	23	0
Tmem cells colon	127	3689	1	4	10	0	6	0
Treg cells skin	1	0	154	0	0	0	1	0
Tmem cells skin	0	1	163	1	1	0	0	0
Treg cells mLN	0	0	0	4309	87	0	32	6
Tmem cells mLN	0	0	0	176	4359	618	24	2
Treg cells bLN	0	0	0	4785	35	0	80	5
Tmem cells bLN	0	1	0	88	2852	248	17	2
Treg cells spleen	0	0	0	3915	40	0	20	8
Tmem cells spleen	0	0	0	47	2637	1875	25	12

Figure S1 (Related to Figure 1) - Sorting, clustering, and identification of Treg and Tmem cells.

(A) Flow cytometry-sorting strategy for sorting Treg and Tmem cells from (top) lymphoid (mLN) and (bottom) non-lymphoid (colonic lamina propria, cLP, as an example) organs. (B) tSNE projection of all 10x dataset cells passing QC, coloured by the resulting graph-based clustering. Cells from the NKT, Stress/Mt and Undefined clusters were removed from further analysis. (C) Number of cells from each cluster in (D) originating from each sorted population. (D and E) Treg and Tmem cells were obtained with the same methodology as in Figure 1A, sequenced using Smart-seq2. t-SNE dimensionality reduction represents all sorted cells for each individual batch that passed quality control (see Methods). Colors match cell-type and tissue of origin. (F) Genes defining the identity of Treg and Tmem cells in lymphoid and non-lymphoid tissues, obtained from the Smart-seq2 datasets. Colon and skin were individually compared with their corresponding draining lymph node and spleen cells. Significantly expressed genes in each cell-type-tissue combination have an average log fold-change greater than 0.25 and and adjusted p-value lower than 0.05 (Wilcoxon test).

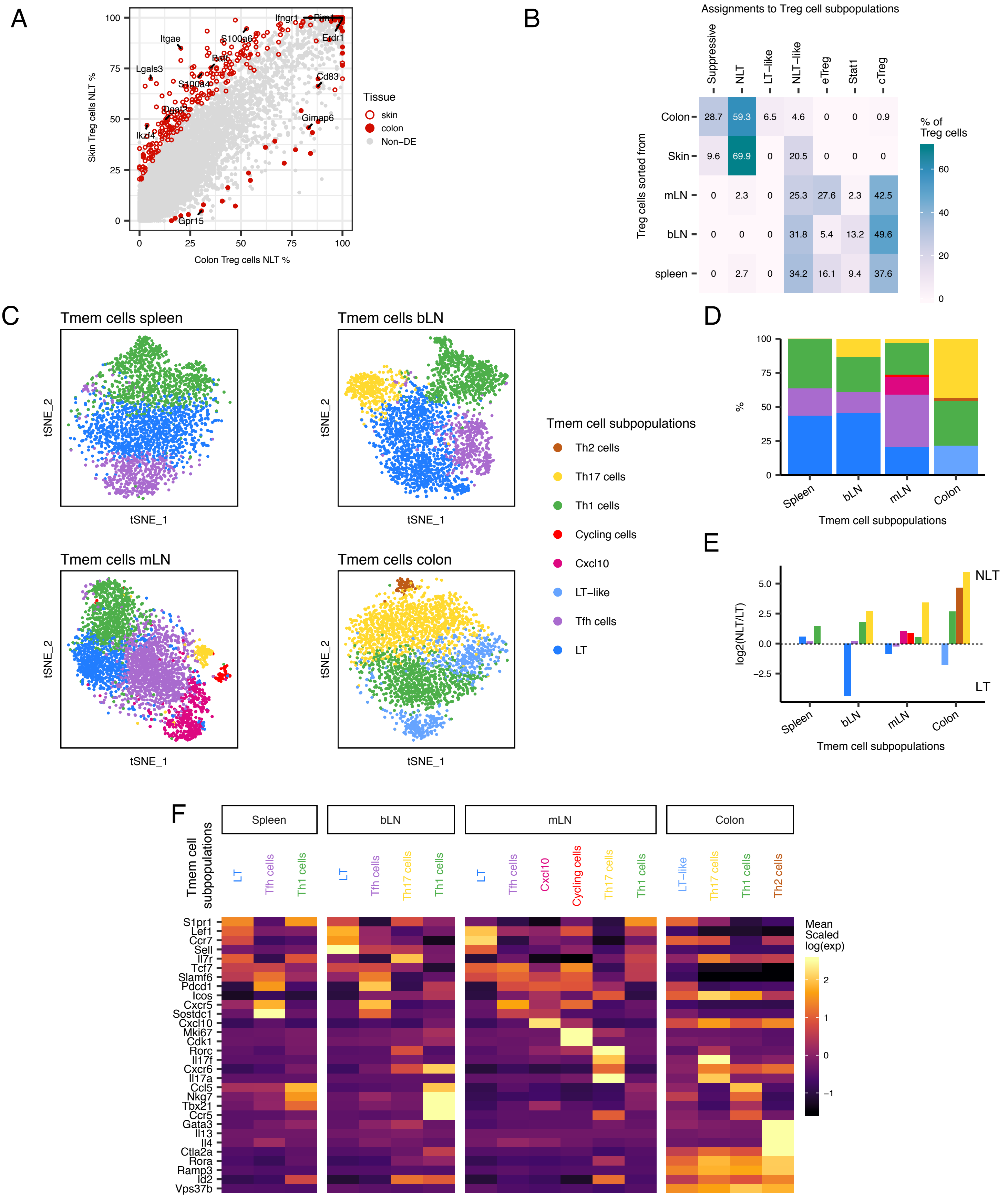

Figure S2 (Related to Figure 2) - Heterogeneity in SS2 and Tmem cell LT and NLT populations.

(A) Percentage of cells expressing each gene in skin Treg NLT and colon Treg NLT subpopulations in Smart-seq2 data. Genes that are upregulated in the skin Treg NLT subpopulation (log2(FC)>0.25 and adjusted p-value<0.05) are represented by an open circle, and genes upregulated in colon Treg NLT (log2(FC)<(-0.25) and adjusted p-value<0.05) are represented by a filled circle. (B) Matching of Smart-seq2 Treg cells sorted populations to identified Treg subpopulations in the 10x dataset using a logistic regression model (85% accuracy, see Methods). Table shows the percentage of each sorted population (y-axis) that were labelled as each Treg cluster (x-axis). (C) t-SNE projection of Tmem cells per tissue coloured by subpopulations found using graph-based clustering. (D) Subpopulation marker gene mean expression levels (z-score) per subpopulation. Gene markers exhibit |log2(FC)|>0.25 and adjusted p-value<0.05 in the comparison of each subpopulation versus all the other cells within the same tissue. Values greater than 2.5 or lower than -1.5 are coloured equally. (E) Relative proportions of Tmem subpopulations within each tissue that revealed heterogeneity. (F) Measure of the NLT/LT signature score in each Tmem subpopulation, measured as the ratio between the number of NLT and LT genes that have been identified as significantly upregulated in each cluster.

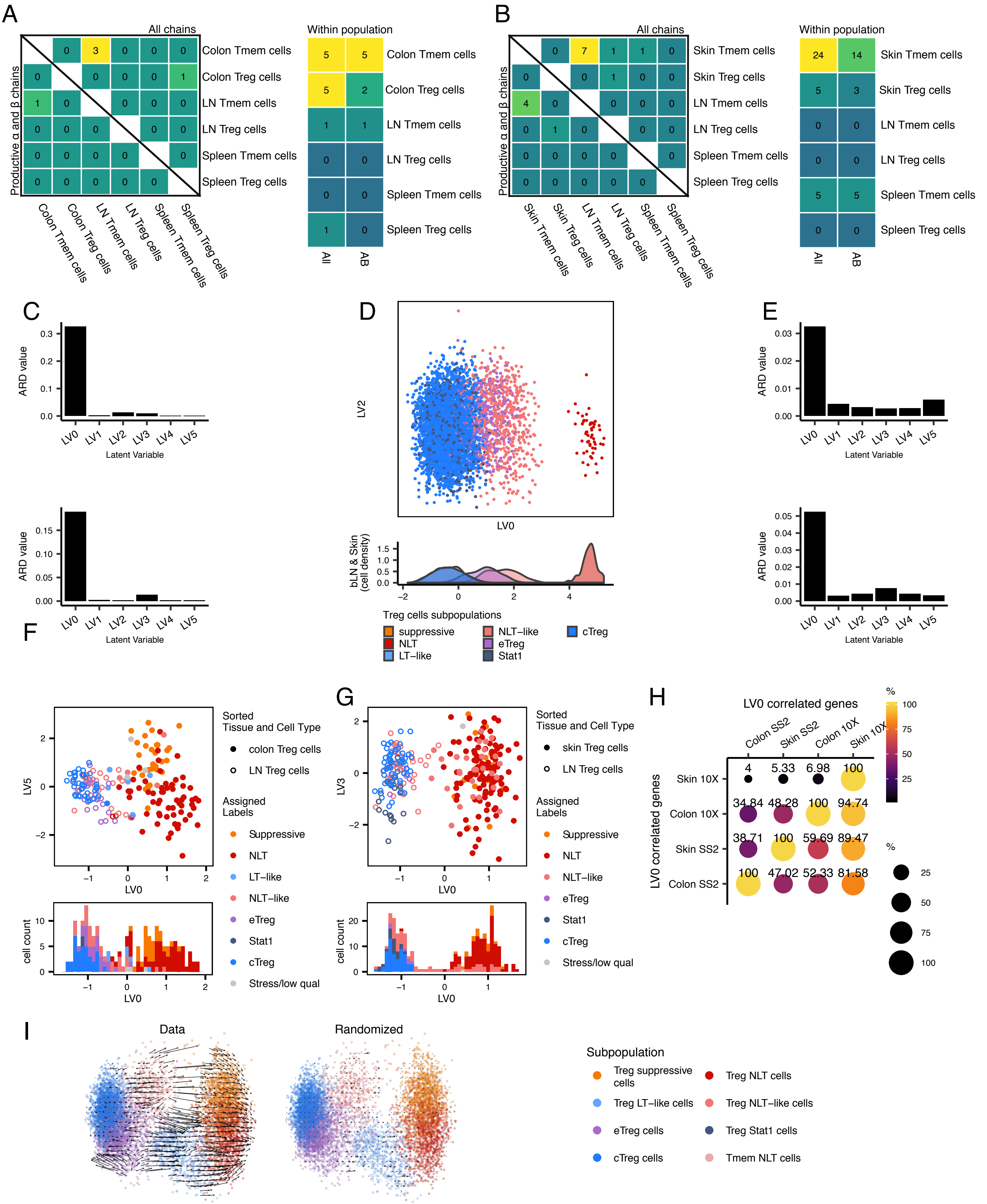

Figure S3 (Related to Figure 3) - Additional evidence on LN-to-NLT transition in the colon and skin datasets.
(A and B) Clonotypes detected using TraCeR in the Smart-seq2 (A) Mouse Colon dataset, or (B) Mouse Skin dataset. In each panel, on the left, number of clonotypes detected spanning different tissues and cell type combinations. Top right half registers all events of TCR chain sharing, bottom left half only considers the sharing of productive α and β TCR chain, and on the right, number of clonotypes detected within each cell type and tissue, considering the sharing of any chain or productive α and β. (C) Automatic Relevance Determination (ARD) plots for BGPLVM of Treg in mLN and colon (top, referring to Figure 3A), and bLN and skin (bottom, referring to Supplementary Figure 6B) datasets. These plots show the relevance of each latent variable extracted from the data. (D) BGPLVM dimensionality reduction of bLN and skin Treg cells from the 10X dataset (top), with a density plot showing the distribution along LV0 of each identified subpopulation (bottom). (E) Automatic Relevance Determination (ARD) plots for BGPLVM of Smart-seq2 Treg in mLN and colon (top, referring to Figure S3F), and bLN and skin (bottom, referring to Figure S3G) datasets. (F and G) BGPLVM dimensionality reduction of Smart-seq2 data of Treg from lymph nodes and non-lymphoid tissues (top), with a histogram plot showing the distribution along LV0 of each subpopulation identified (bottom). mLN and colon Treg are considered in (E), while bLN and skin Treg in (F). Cells are coloured by the inferred subpopulation they belong to as per the predictions made in Figure S2B. (H) Pairwise overlap between the sets of genes with absolute correlation with LV0 greater than 0.25 in each of the four steady-state datasets. The percentages refer to the proportion of the set on the x-axis that is overlapping the set on the y-axis. (I) Velocyto vectorfield overlaid on BGPLVM projection of mLN and colon Treg cells (related to Figure 3A).

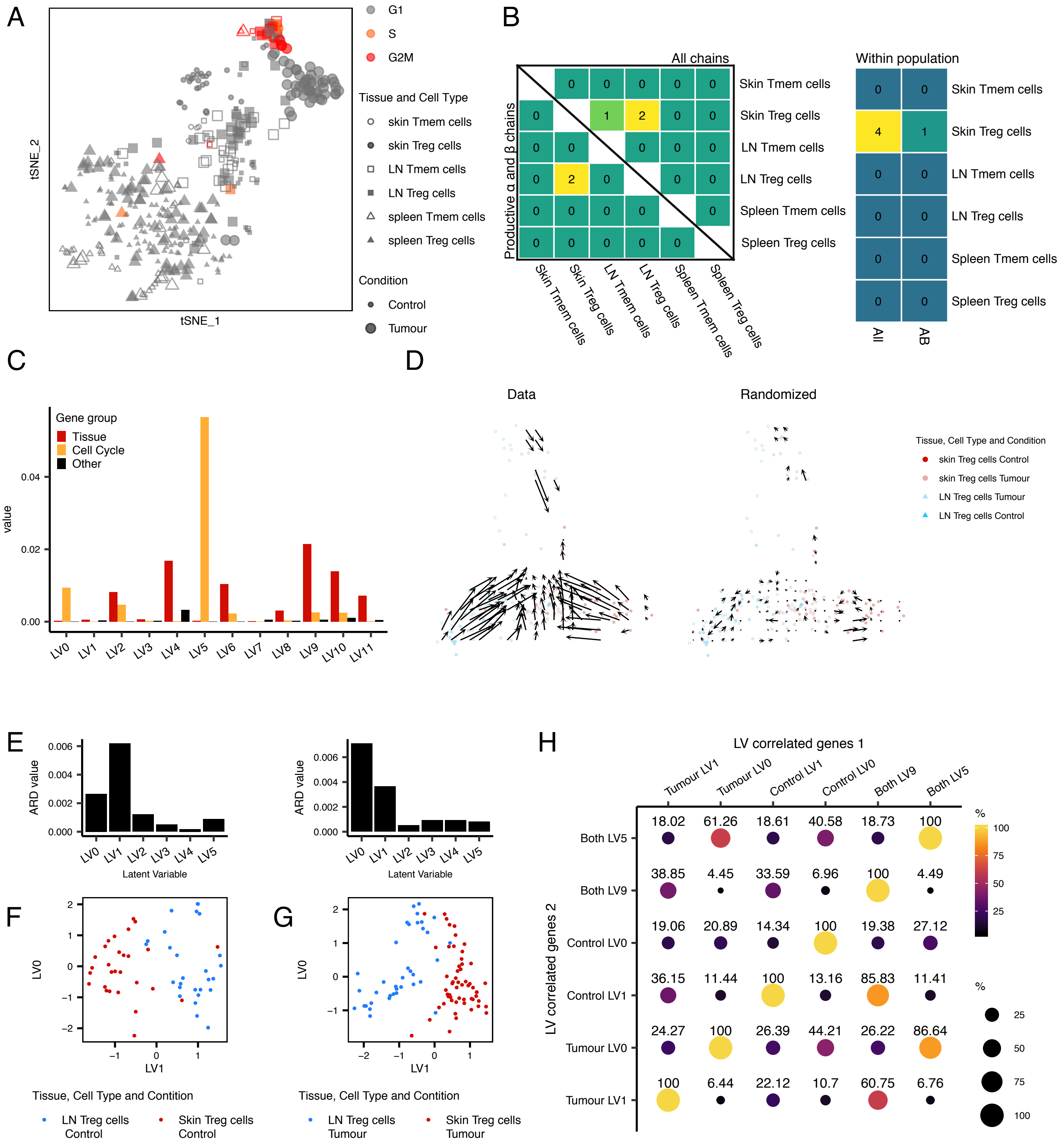

Figure S4 (Related to Figure 4) - Additional details on the MRD-BGPLVM projection.

(A) t-SNE dimensionality reduction coloured by cell cycle phase in the mouse melanoma dataset. (B) Clonotypes detected using TraCeR in the Smart-seq2 Mouse Melanoma dataset. In each panel, on the left, number of clonotypes detected spanning different tissues and cell type combinations. Top right half registers all events of TCR chain sharing, bottom left half only considers the sharing of productive α and β TCR chain, and on the right, number of clonotypes detected within each cell type and tissue, considering the sharing of any chain or productive α and β. (C) Automatic Relevance Determination (ARD) plots for MRD-BGPLVM of Treg in control and melanoma conditions. Colours show effect of gene groups in each obtained latent variable. (D) Velocyto vectorfield overlaid on MRD-BGPLVM projection of bLN and skin from both Control and Melanoma conditions (related to Figure 4D). (E) Automatic Relevance Determination (ARD) plots for BGPLVM of Smart-seq2 Treg in bLN and skin in the Control condition (left, related to Figure S4F), and Melanoma condition (bottom, related to Figure S4G). (F and G) BGPLVM projection of bLN and skin in control (F) and melanoma (G) conditions, using the top two latent variables. (H) Pairwise overlap between the sets of genes with absolute correlation with LV0 greater than 0.25 in each subset of the melanoma dataset. The percentages refer to the proportion of the set on the x-axis that is overlapping the set on the y-axis.

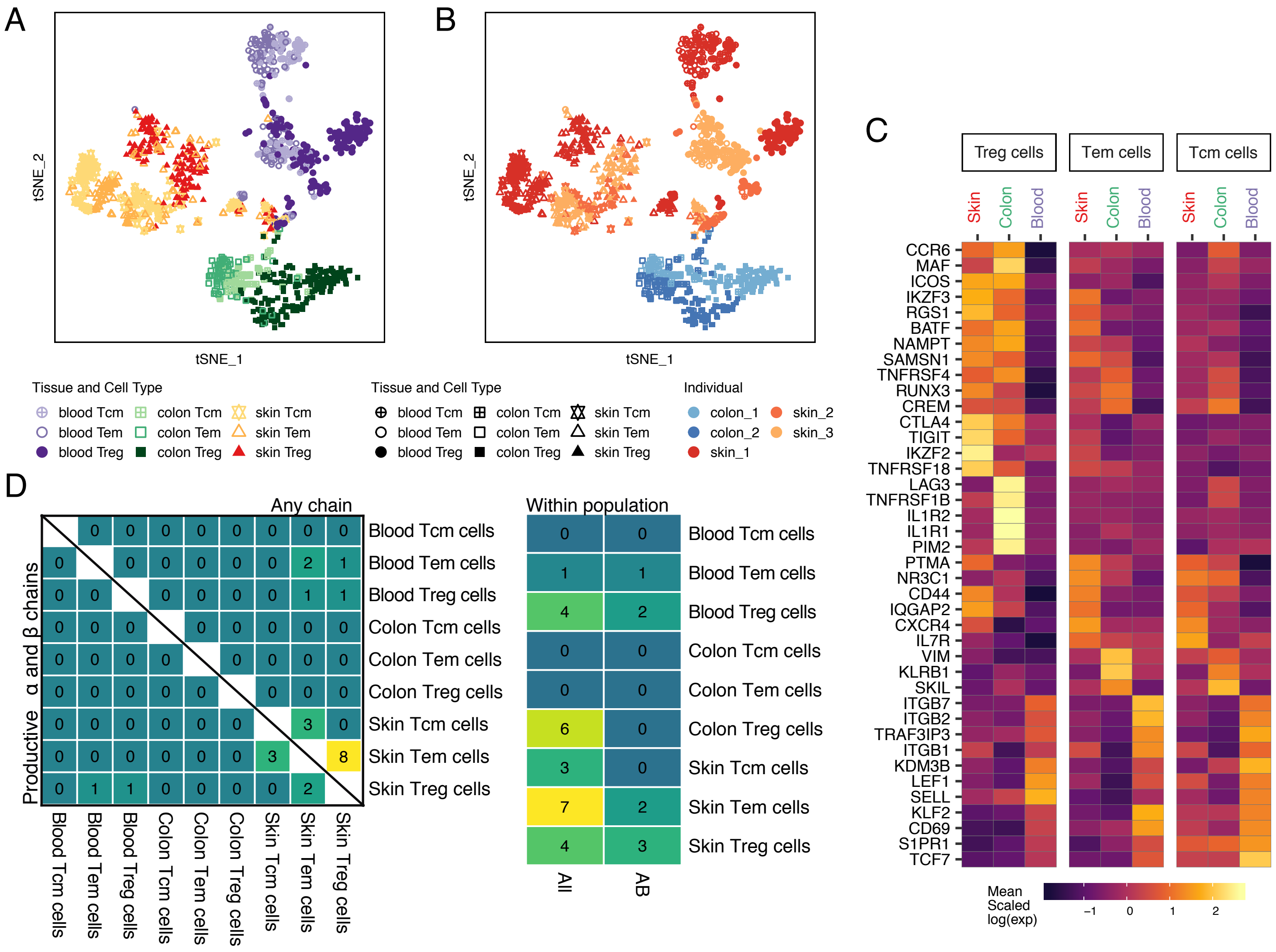

Figure S5 (Related to Figure 5) - Additional information about the Human Smart-seq2 dataset.

(A and B) t-SNE dimensionality reduction. Shapes match cell type and tissue according to legend. Colours match either cell type and tissue (A) or sampled individual (B). (C) Z-score of mean expression levels of identified markers across all sampled cell types and tissues in human. (D) Clonotypes detected using TraCeR in the Smart-seq2 Human dataset. In each panel, on the left, number of clonotypes detected spanning different tissues and cell type combinations. Top right half registers all events of TCR chain sharing, bottom left half only considers the sharing of productive α and β TCR chain, and on the right, number of clonotypes detected within each cell type and tissue, considering the sharing of any chain or productive α and β.
